# Supplementary figures and images for: The nucleoid as a scaffold for the assembly of bacterial signaling complexes
Source: PLoS Genet. 2017 Nov 21;13(11):e1007103. doi: 10.1371/journal.pgen.1007103 (PMC5716589; doi:10.1371/journal.pgen.1007103)

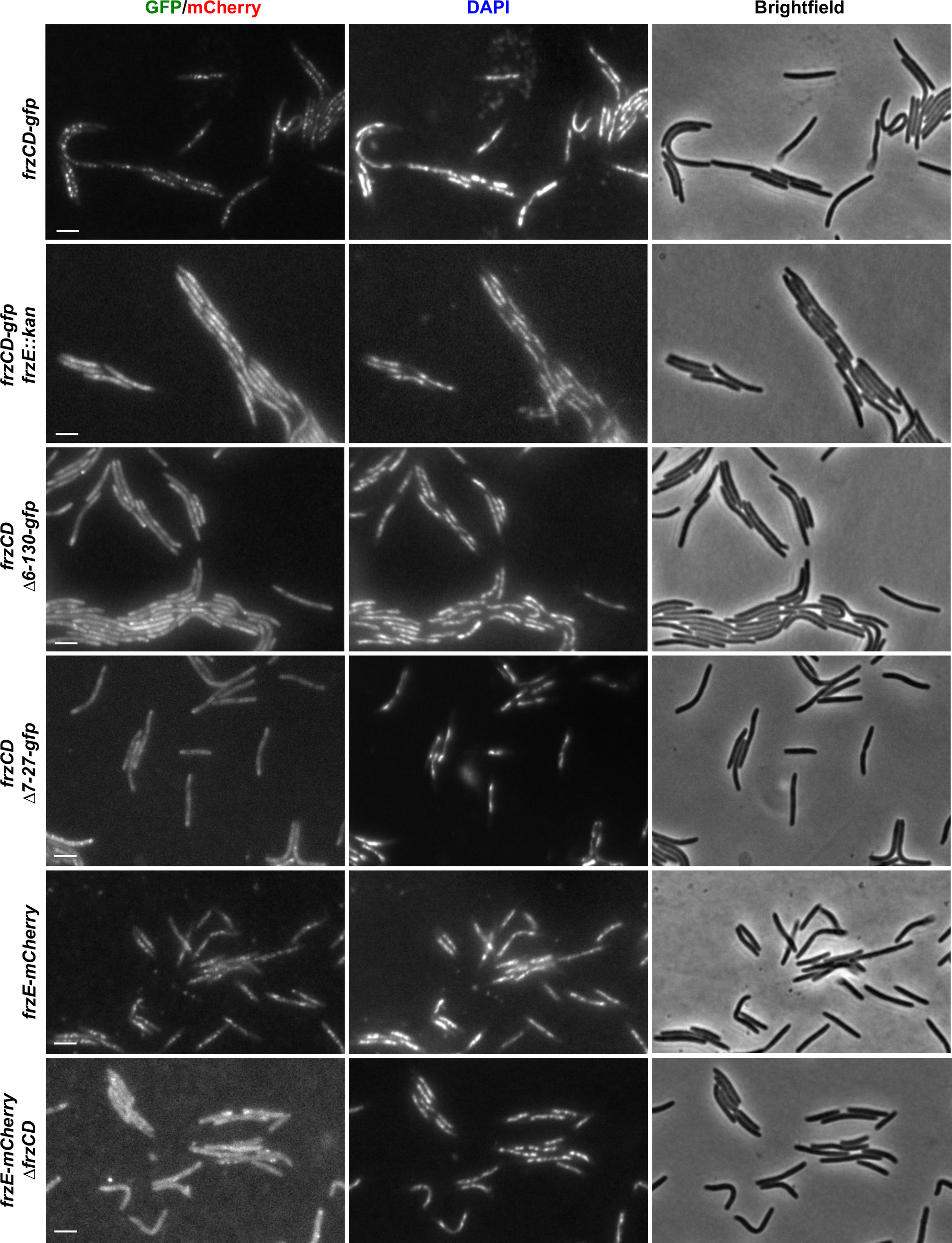

Supplement: S1 Fig — The genetic backgrounds of the M. xanthus strains are indicated on the left. Scale bars correspond to 1μm. (TIF) [file pgen.1007103.s001.tif]

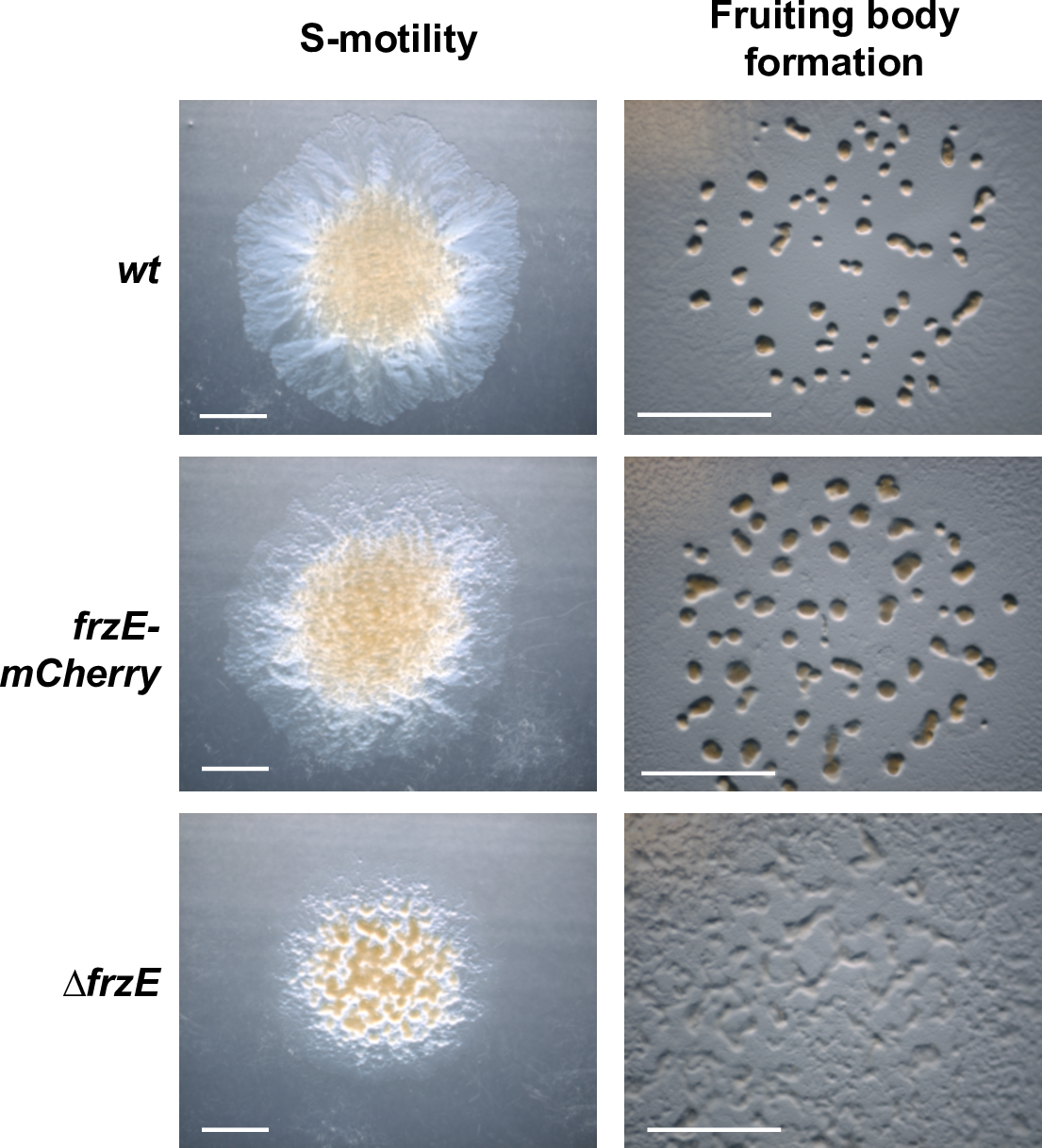

Supplement: S2 Fig — Scale bars correspond to 0,5 cm. (TIF) [file pgen.1007103.s002.tif]

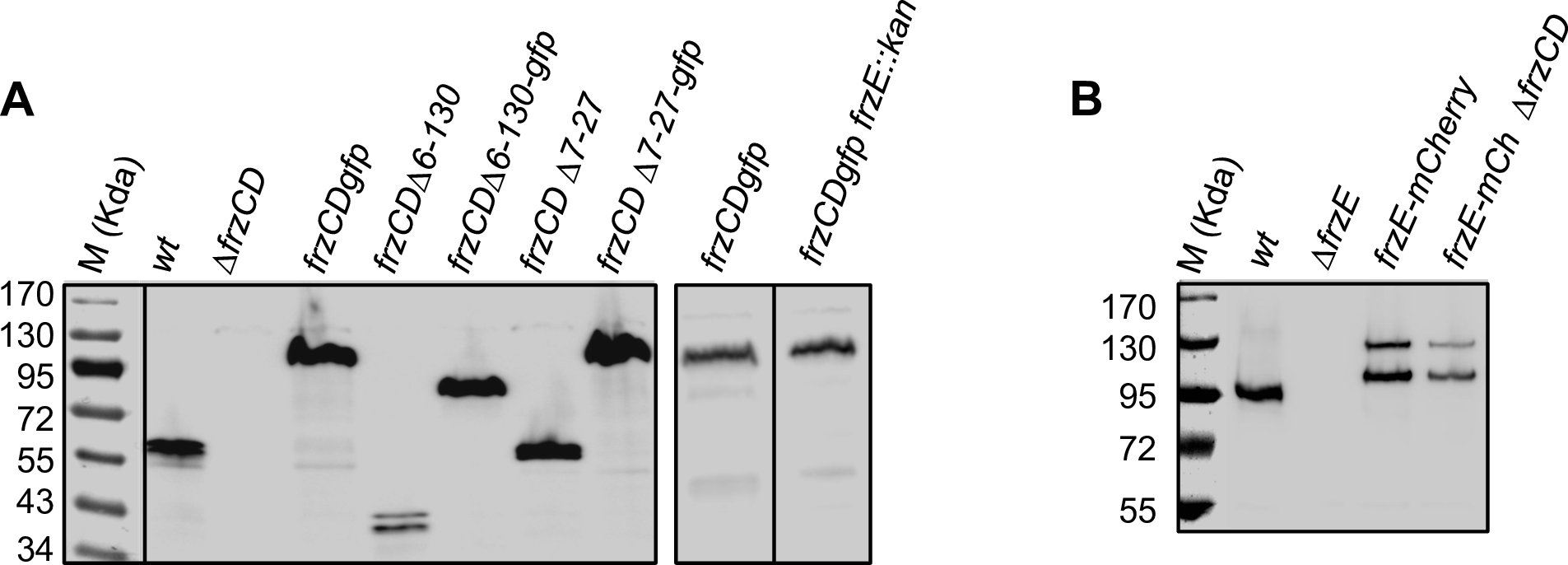

Supplement: S3 Fig — Western blot with anti-FrzCD (A) or anti-FrzE antibodies (B) on the cell extracts of the indicated M. xanthus strains. Black lines are used to indicate that two lanes from the same gel where separated by other lanes in the original western blot. (TIF) [file pgen.1007103.s003.tif]

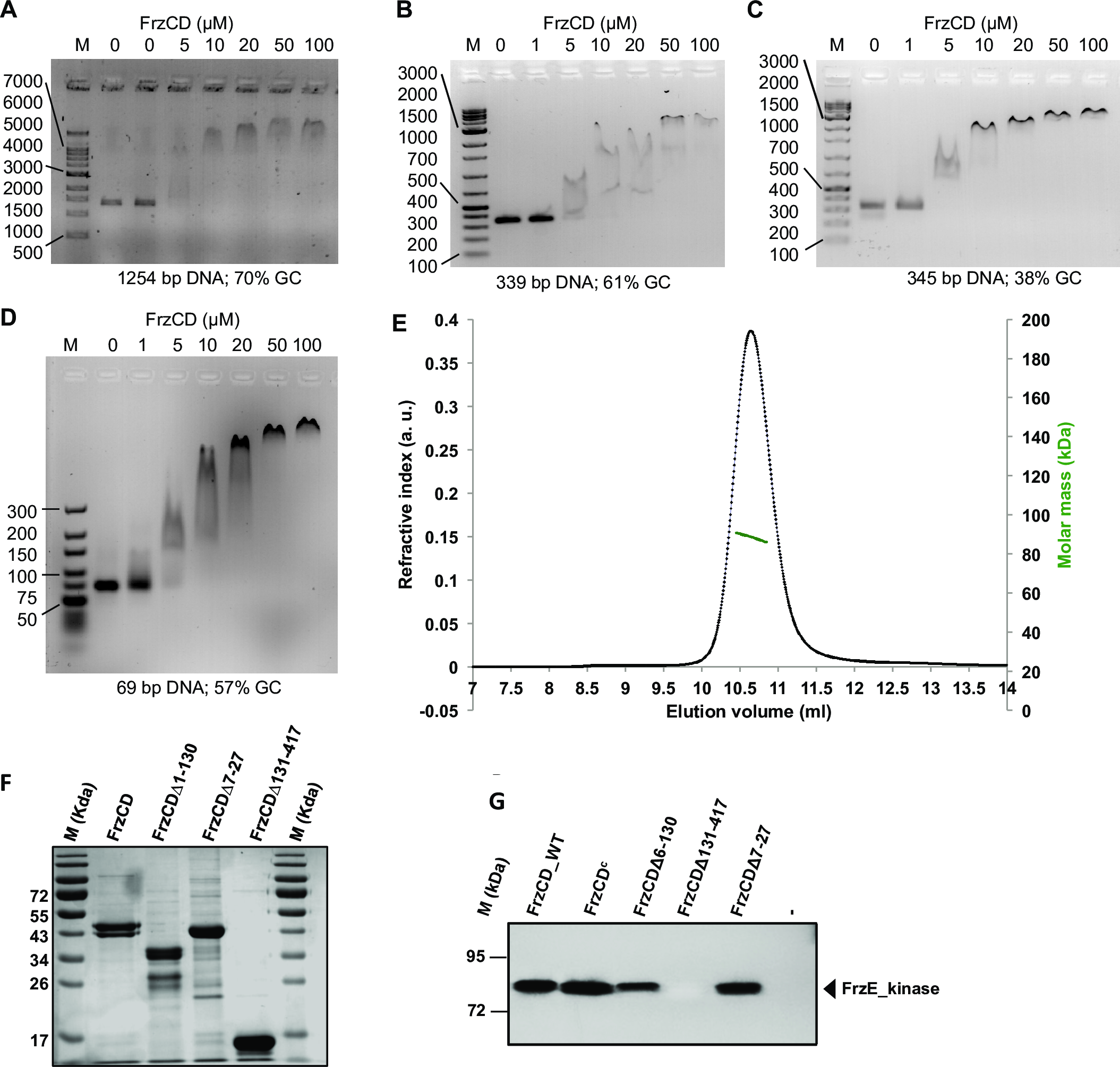

Supplement: S4 Fig — (A- D) Electrophoretic mobility shift assay (EMSA) on agarose gel stained with ethidium bromide and developed at the UV light. DNA fragments of different lengths and GC contents (S4 Table) were incubated with increasing concentrations of FrzCD-(His6) as indicated, including (A) 4 nM of Oligo1300 (1% agarose gel), (B) 40 nM of Oligo340-1 (38% GC content; 1.5% agarose gel), (C) 40 nM of Oligo340-2 (61% GC content; 1.5% agarose gel), and (D) 300 nM of Oligo70 (2.5% agarose gel). (E) SEC-MALS analysis showing the elution profile (refractive index; black; left y-axis) and the estimated molar masses (green; right y-axis) of the FrzCD eluted protein. (F) SDS page of the indicated proteins purified from E. coli and used for the different experiments shown in Fig 3. (G) Kinetics of the FrzE kinase domain (FrzECheA) auto-phosphorylation were tested in vitro by incubation of FrzECheA in the presence of FrzA, the indicated different form of FrzCD and ATPγP33 as a phosphate donor. (TIF) [file pgen.1007103.s004.tif]

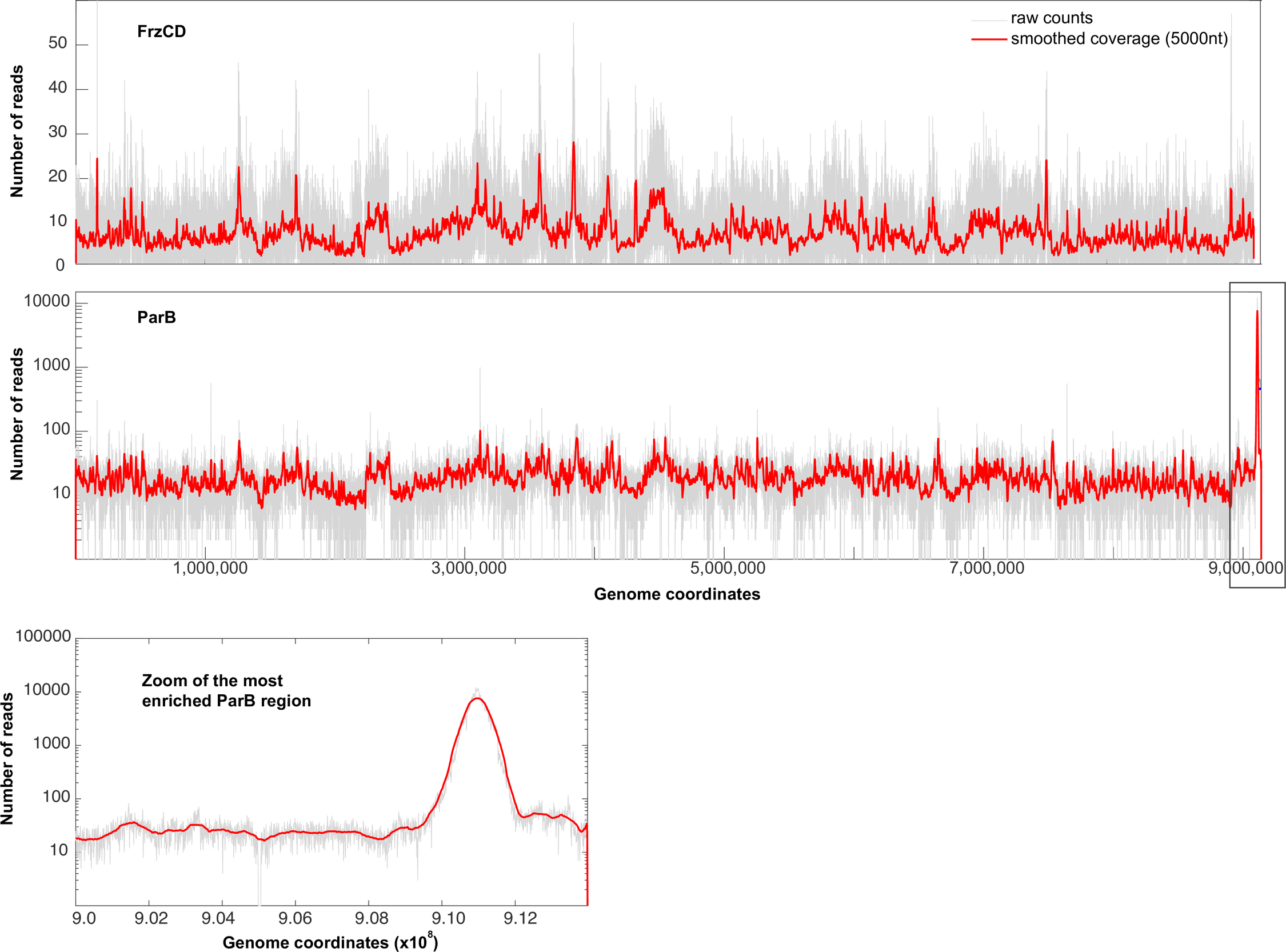

Supplement: S5 Fig — A library of DNA fragments was obtained by ChIP experiments on frzCD-gfp and parB-yfp strains, using GFP polyclonal antibodies. The figure shows the results obtained by the deep sequencing of the DNA libraries. Only for parB-yfp, we observed an enrichment corresponding to the nucleoid region containing parS (rectangle in the middle panel and last panel) (9,109 to 9,110 Kb). Note that while the number of reads relative to ParB are represented with a logarithmic scale, for FrzCD we used a regular scale. (TIF) [file pgen.1007103.s005.tif]

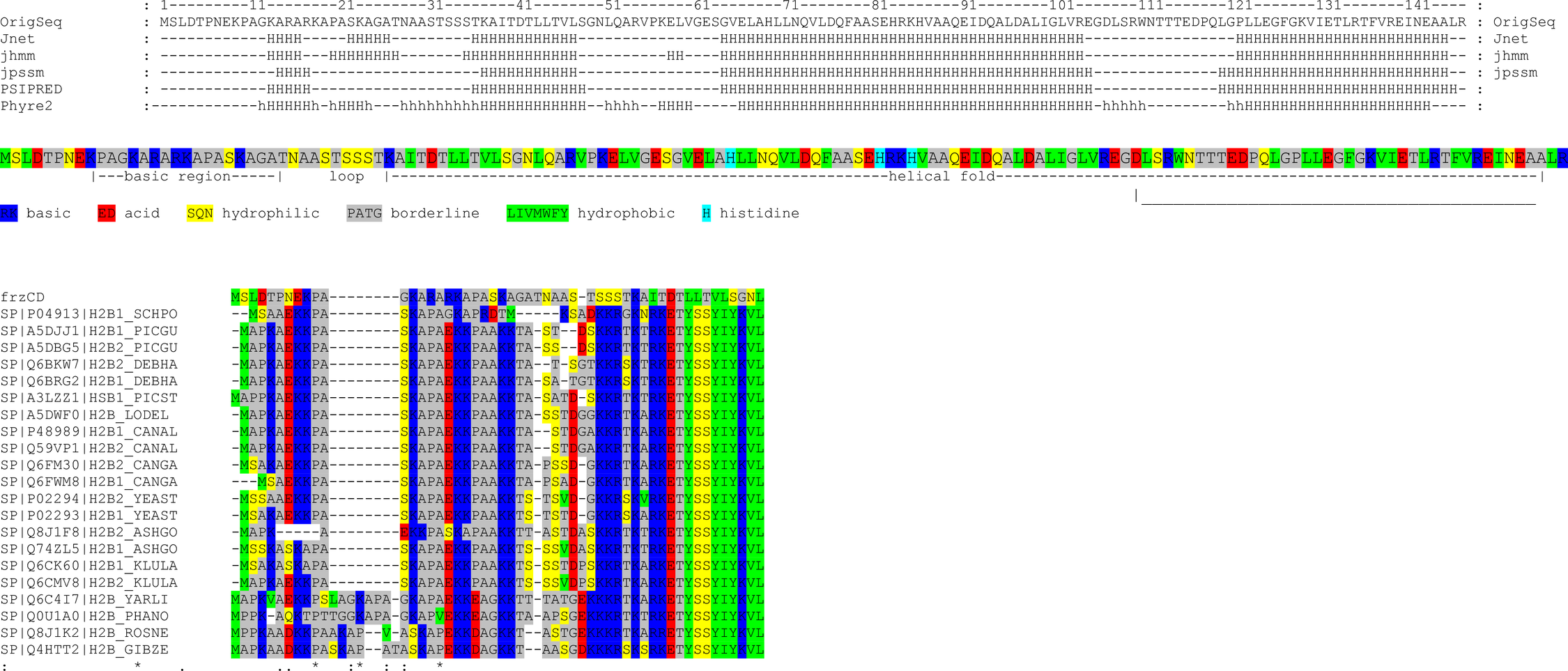

Supplement: S6 Fig — On the top, the prediction of the FrzCD N-terminal secondary structures is shown. The nature of each amino acid is also indicated through color codes. The bottom part of the figure shows the FrzCD first 50 amino acid alignment with the N-terminal tail of Histones 2B. The alignment was obtained by Clustal Omega. Dots indicate similarities and stars identities. (TIF) [file pgen.1007103.s006.tif]

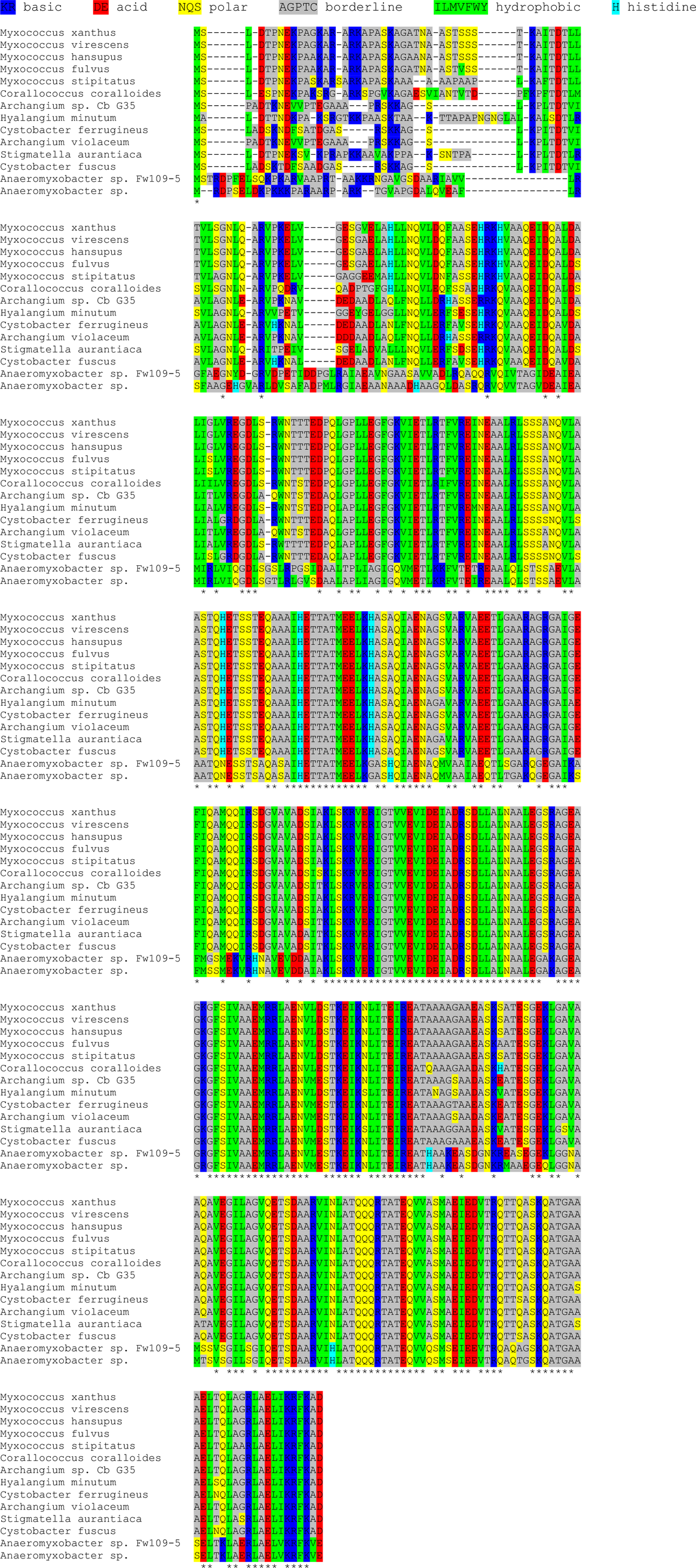

Supplement: S7 Fig — The alignment of FrzCD sequences from the indicated species was obtained by Clustal Omega. Stars indicate identities. (TIF) [file pgen.1007103.s007.tif]
