# Supplementary material for: The nucleoid as a scaffold for the assembly of bacterial signaling complexes
Source: PLoS Genet. 2017 Nov 21;13(11):e1007103. doi: 10.1371/journal.pgen.1007103 (PMC5716589; doi:10.1371/journal.pgen.1007103)
Supplement: S1 Table — (DOCX) [file pgen.1007103.s008.docx]

S1 Table

| Strains | Genotype | Deletion | Source |
| --- | --- | --- | --- |
|  |  |  |  |
| DZ2 | *wt* |  | Zusman et al., 1982 |
| DZ4620 | *frzCD-gfp* |  | Mauriello et al., 2009 |
| DZ4480 | *∆frzCD* | Codons 6-393 | Bustamante et al., 2004 |
| DZ4485 | *frzCD^∆6-130^* |  | Mauriello et al., 2009 |
| DZ4743 | *frzCD^∆6-130^-gfp* |  | Mauriello et al., 2009 |
| EM231 | *frzCD_E202A-E203A_::gfp* |  | Mauriello et al., 2009 |
| EM228 | *frzCD_E168A-G169A_::gfp* |  | Mauriello et al., 2009 |
| EM434 | *frzE-mCherry* |  | This study |
| EM506 | *frzE-mCherry ∆frzCD* |  | This study |
| EM516 | *frzCD-gfp frzE::kan* | Codons 171-438 | This study |
| EM531 | *difA-gfp ∆parB/P_cuoA_-parB* |  | This study |
| EM532 | *frzCD-gfp ∆parB/P_cuoA_-parB* |  | This study |
| EM533 | *frzE-mCherry ∆parB/P_cuoA_-parB* |  | This study |
| EM543 | *frzCD^∆7-27^* | Codons 7-27 | This study |
| EM550 | *frzCD^∆7-27^-gfp* | Codons 7-27 | This study |
| TM26 | *frzS-yfp* |  | Guzzo et al., 2015 |
| EM622 | *frzCD^∆6-130^ frzS-yfp* |  | This study |
| EM623 | *∆frzCD frzS-yfp* |  | This study |
