# Supplementary material for: The nucleoid as a scaffold for the assembly of bacterial signaling complexes
Source: PLoS Genet. 2017 Nov 21;13(11):e1007103. doi: 10.1371/journal.pgen.1007103 (PMC5716589; doi:10.1371/journal.pgen.1007103)
Supplement: S2 Table — (DOCX) [file pgen.1007103.s009.docx]

S2 Table

| Plasmid | Description | Source |
| --- | --- | --- |
|  |  |  |
| pAH57 | *P_cuoA_-parB, c*opper-dependent expression of *parB*, Mx8 attB | Harms et al., 2013 |
| pAH18 | Construct for in frame deletion of *parB* | Harms et al., 2013 |
| pETPhos_*frzCD* | pETPhos with *frzCD* tagged with 6-his inducible with IPTG | Guzzo et al., 2015 |
| pETPhos_*frzE^kinase^* | pETPhos with *frzE^CheA^* tagged with 6-his inducible with IPTG | Guzzo et al., 2015 |
| pETPhos_*frzCD^c^* | pETPhos with *frzCD^c^* tagged with 6-his inducible with IPTG | Guzzo et al., 2015 |
| pGEX(M)_*frzA* | pETPhos with *frzA* tagged with 6-his inducible with IPTG | Guzzo et al., 2015 |
| pEM365 | pBJ113 with frzE-mCherry fusion | This study |
| pEM405 | pBJ113 with an insertion cassette for *frzE::kan* | This study |
| pEM418 | pBJ113 with a cassette for *frzCD^∆7-27^* | This study |
| pEM414 | pETPhos with *frzCD^∆1-130^* tagged with 6-his inducible with IPTG | This study |
| pEM415 | pETPhos with *frzCD^∆131-417^* tagged with 6-his inducible with IPTG | This study |
| pEM433 | pETPhos with *frzCD^∆7-27^* tagged with 6-his inducible with IPTG | This study |
| pEM409 | pETDuet-1 with *frzCD-gfp* inducible with IPTG | This study |
| pEM417 | pETDuet-1 with *frzCD^∆1-130^-gfp* inducible with IPTG | This study |
| pEM434 | pETDuet-1 with *frzCD^∆7-27^-gfp* inducible with IPTG | This study |
| pEFrzSY | pEYFPN1 with a cassette to construct *frzS-yfp* | Guzzo et al., 2015 |
| pFCD-H6 | pHis17 with *frzCD* gene tagged with a C-terminal 6-his | This study |
