# Supplementary material for: The nucleoid as a scaffold for the assembly of bacterial signaling complexes
Source: PLoS Genet. 2017 Nov 21;13(11):e1007103. doi: 10.1371/journal.pgen.1007103 (PMC5716589; doi:10.1371/journal.pgen.1007103)
Supplement: S3 Table — (DOCX) [file pgen.1007103.s010.docx]

S3 Table

| %IAA  Strain | **0** | **0.005** | **0.01** | **0.03** | **0.05** | **0.1** | **0.3** |
| --- | --- | --- | --- | --- | --- | --- | --- |
| ***frzS-yfp*** | 101 | 113 | 77 | 114 | 64 | 47 | 170 |
| ***frzCD^∆6-130^ frzS-yfp*** | 98 | 103 | 75 | 128 | 112 | 139 | 109 |
| ***∆frzCD frzS-yfp*** | 44 | ND | ND | ND | 48 | ND | 47 |
