# Supplementary material for: The nucleoid as a scaffold for the assembly of bacterial signaling complexes
Source: PLoS Genet. 2017 Nov 21;13(11):e1007103. doi: 10.1371/journal.pgen.1007103 (PMC5716589; doi:10.1371/journal.pgen.1007103)
Supplement: S4 Table — (DOCX) [file pgen.1007103.s011.docx]

S4 Table

| Ds DNA | Sequence (5’ 🡪 3’) | Length,  GC content (%) |
| --- | --- | --- |
|  |  |  |
| Oligo70 | CTTGCAGTAGAGCTGACCATGATTACGCCATCAGCAGCTCCAGGTCGTACCTCCAGCTACCAATCCCCG | 69, 57 |
| Oligo340-1 | TAATACGACTCACTATAGGGAGACCACAACGGTTTCCCTCTAGAAATAAT  TTTGTTTAACTTTAAGAAGGAGATATACATATGTCCCTGGACACCCCCAA  CGAGAAGCCCGCTGGCAAGGCTCGCGCCCGGAAGGCCCCCGCCTCCAAGG  CCGGCGCCACGAACGCGGCGTCGACCTCTTCCTCCACCAAGGCCATCACC  GACACGCTGCTGACGGTGCTGTCCGGCAACCTGCAGGCCCGCGTGCCCAA  GGAGCTGGTCGGTGAGTCCGGCGTGGAGCTGGCGCACCTGCTCAACCAGG  TGCTGGACCAGTTCGCGGCCTCCGAGCACCGCAAGCATG | 339, 61 |
| Oligo340-2 | GTTTAACTTTAAGAAGGAGATATACATATGAAAAAAGAAACGATTTTTTCCGAAGTAGAAACGGCTAACAGCAAGCAACTGGCTGTGTTGAAAGCTAATTTCCCACAGTGTTTTGATAAAAACGGAGCCTTCATTCAAGAAAAATTGCTTGAGATTATTAGGGCATCGGAAGTTGAACTCTCTAAAGAATCATACAGCTTGAACTGGCTGGGTAAATCTTATGCCCGTTTGTTGGCCAATCTACCACCGAAAACGTTGTTGGCAGAAGATAAAACTCATAACCAACAAGAAGAGAACAAGAACAGTCAACACCTGTTAATCAAAGGGGATAATCTCGAAGTATTG | 345, 38 |
| Oligo1300 | *frzCD* gene amplified with the primers  5’-ATGTCCCTGGACACCCCCAACGAGAAGCCCGCTGG-3’and  5’-CTAGTCGGCCTTGAACCGCTTGATGAGCTCGGCCA-3’ and the plasmid pFCD-H6 as template | 1254, 70 |
| Oligo801 | Fragment amplified with the primers  5’-cgggatcctggctccgcccccgacgca-3’ and  5’-cccaagcttttgatgaggcgcttggagat-3’  and DZ2 chromosomal DNA as template | 801, 68 |
